# Supplementary material for: Engineering probiotics to inhibit Clostridioides difficile infection by dynamic regulation of intestinal metabolism
Source: Nat Commun. 2022 Jul 4;13:3834. doi: 10.1038/s41467-022-31334-z (PMC9253155; doi:10.1038/s41467-022-31334-z)
Supplement: Supplementary file 1 — Supplemental Information [file 41467_2022_31334_MOESM1_ESM.pdf]

SUPPLEMENTARY MATERIAL

Engineering probiotics to inhibit *Clostridioides difficile* infection by dynamic regulation of intestinal metabolism

Elvin Koh[1,2,3 ‡], In Young Hwang[1,2,3 ‡], Hui Ling Lee[1,2,3], Ryan De Sotto[1,2,3], Jonathan W. J. Lee[1,2,4], Yung Seng Lee[1,2,5], John C. March[6], Matthew Wook Chang[1,2,3 *]

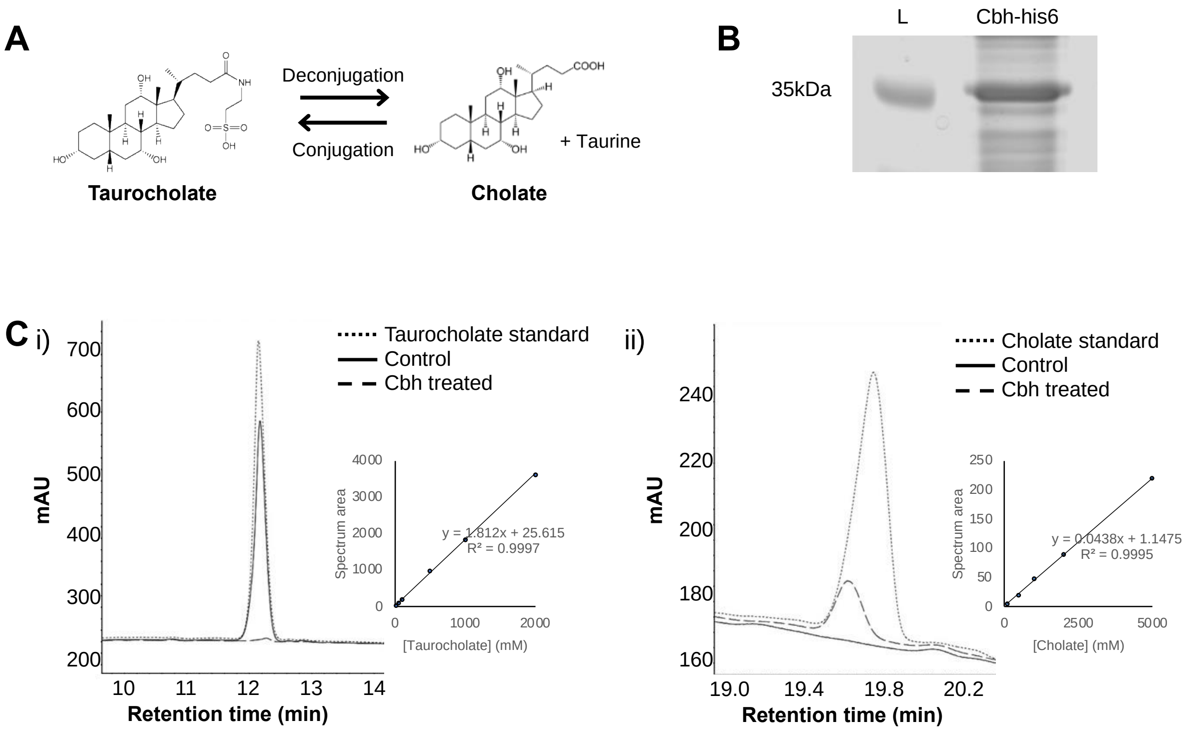

Supplementary Figure 1: Effect of taurocholate and cholate on *C. difficile*. A) Chemical structures of the conjugated bile salt taurocholate and the primary bile salt cholate. Taurocholate is converted into cholate via deconjugation. B) SDS-PAGE resolution of purified Cbh-his6 (bile salt hydrolase) from the *E. coli* BL21 strain. The expected size of Cbh-his6 is 38 kDa. L represents the protein ladder. The band on the ladder corresponds to 35 kDa. C) HPLC spectra for bile salt quantification following an activity assay for Cbh. Taurocholate was incubated with purified Cbh ("*Cbh treated*") or without the enzyme ("*Control*"). Peaks corresponding to i) taurocholate and ii) cholate were eluted at retention times of 12.2 minutes and 19.6 minutes, respectively. Inserts: standard curves of HPLC spectra generated for taurocholate and cholate.

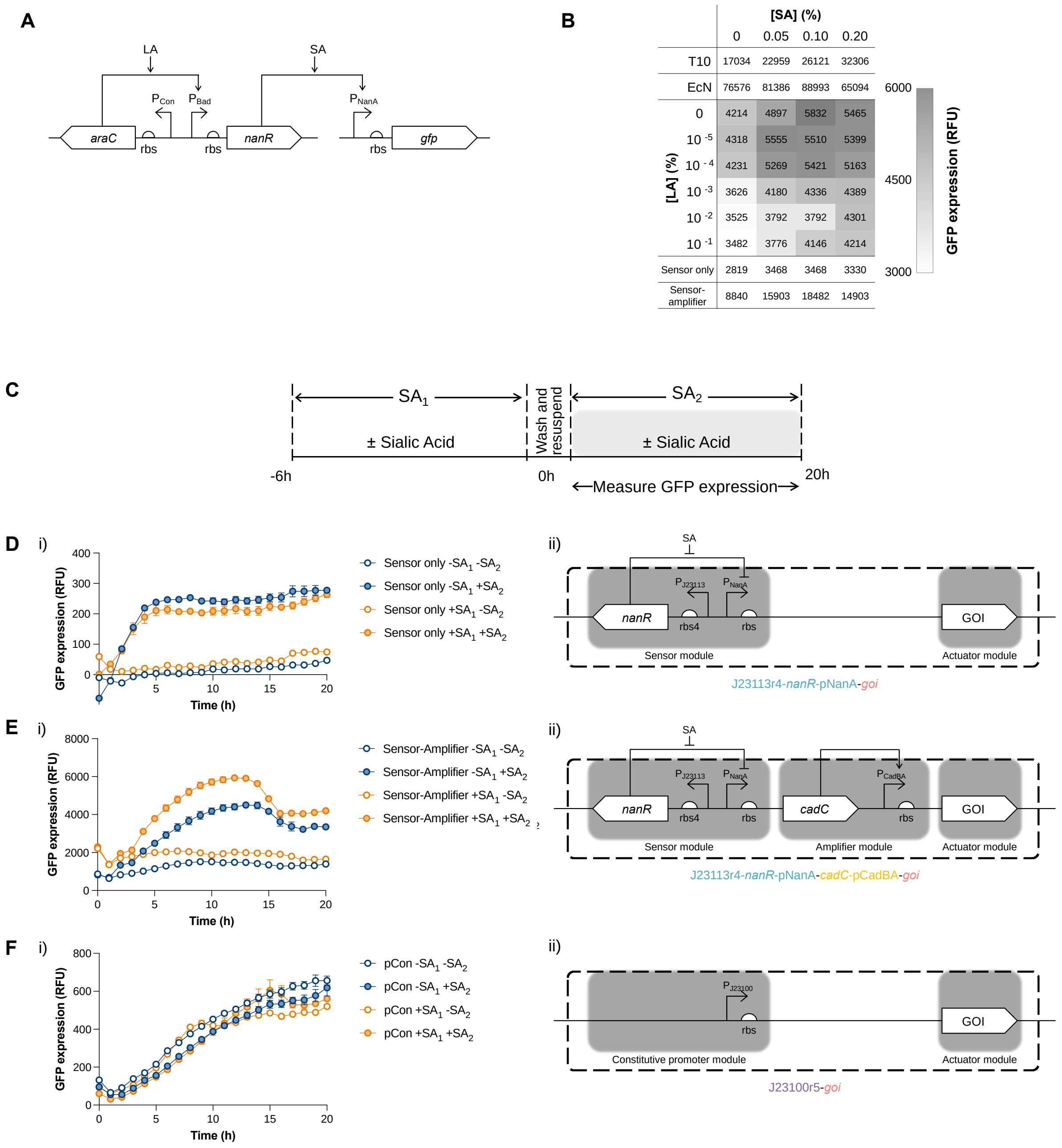

Supplementary Figure 2: Characterisation of the sialic acid-responsive circuit. A) Design of co-expressed plasmids for characterisation of the effect of NanR on pNanA activity. NanR was expressed under the control of pBad in the pSC101-*araC* vector (left), while the GFP reporter was expressed under the control of pNanA in the CoIE vector (right). The levels of inducers were varied to fine-tune the characterisation of the effect of NanR on pNanA activity. LA represents L-arabinose induction. SA represents sialic acid induction. B) Matrix data of the median GFP fluorescence readings of 10,000 size-gated samples from flow cytometry for the following (from top): the expression of pNanA-*gfp* only in Top10, the expression of pNanA-*gfp* only in EcN, the expression of pBad-*nanR* and pNanA-*gfp* in EcN under different combination of sialic acid ("*SA*") and L-arabinose ("*LA*") induction (the colour of each cell is graded to scale), the expression of the *sensor only* construct J23113-*nanR*-pNanA-*gfp* in EcN, and the expression of the *sensor-amplifier* construct J23113-*nanR*-pNanA-*cadC*-pCadBA-*gfp* in EcN.

C) The cells were first induced with sialic acid for 6 hours (+SA_1) or without (-SA_1), followed by the removal of the supernatants and then induced with fresh sialic acid (+SA_2) or without (-SA_2) (shown in graphical representation). i) Relative GFP expression from D) the sensor-only construct (J23113-nanR-pNanA-*gfp*; Sensor only), E) the sensor-amplifier construct (J23113-*nanR*-pNanA-*cadC*-pCadBA-*gfp*; Sensor-Amplifier) and F) the constitutive expression construct (J23100-gfp; pCon) in EcN. The error bars represent the SEMs of triplicates. ii) Circuit diagrams representing respective expression constructs.

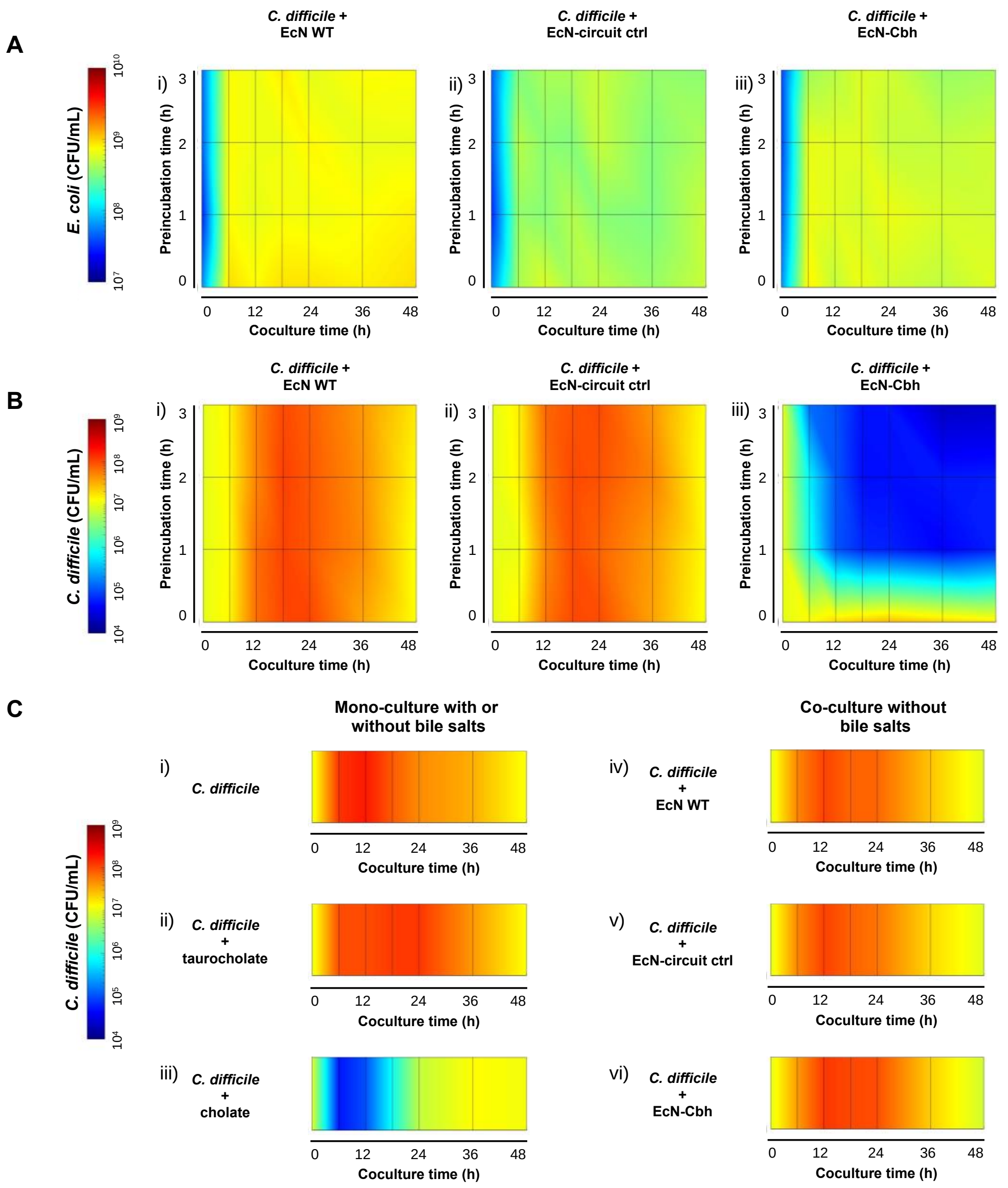

Supplementary Figure 3: *C. difficile* co-culture and mono-culture growth assay. A) Heatmap representation of *E. coli* CFU values from co-culture assays between *C. difficile* and i) wild-type EcN (*EcN WT*), ii) EcN-circuit control (*EcN-circuit ctrl*), or iii) *EcN-Cbh*. The respective *E. coli* were pre-incubated with 2mM taurocholate between 0 and 3 hours and then co-cultured with *C. difficile*. The CFU values were enumerated at regular intervals. Left: logarithm-scaled colour bars corresponding to the CFU/mL values on the heatmap. B) Heatmap representation of *C. difficile* CFU values from co-culture assays between vegetative *C. difficile* cell cultures and i) EcN WT, ii) *EcN-circuit ctrl* or iii) EcN-Cbh. The respective *E. coli* were pre-incubated with taurocholate between 0 and 3 hours and then co-cultured with *C. difficile*. The CFU values were enumerated at regular intervals. Left: logarithm-scaled colour bar corresponding to the CFU/mL values on the heatmap of *C. difficile*. C) *C. difficile* CFU values determined at regular intervals from mono-culture [in i) BHIS growth medium only, ii) BHIS growth medium with taurocholate supplementation, or iii) BHIS growth medium with cholate supplementation] and co-culture in the absence of the bile salt taurocholate or cholate [with i) *E. coli* Nissle wild-type, ii) *EcN-circuit ctrl*, or iii) EcN-Cbh.] Left: logarithm-scaled colour bars corresponding to the CFU/mL values on the heatmap.

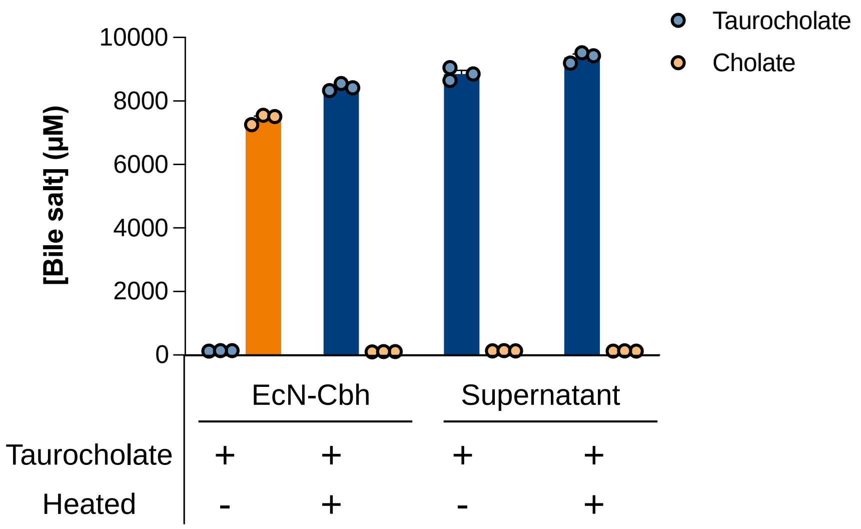

Supplementary Figure 4. Extracellular bile salt deconjugation activity of Cbh. Quantification of taurocholate and cholate after 3 hour incubation of taurocholate with EcN expressing Cbh (EcN-Cbh) or supernatant collected from EcN-Cbh cell culture. Heat treatment (95ºC for 30min) was applied to cells or supernatant prior to incubation with taurocholate, for enzyme inactivation. Data are presented as mean values with error bars representing SEMs of triplicates.

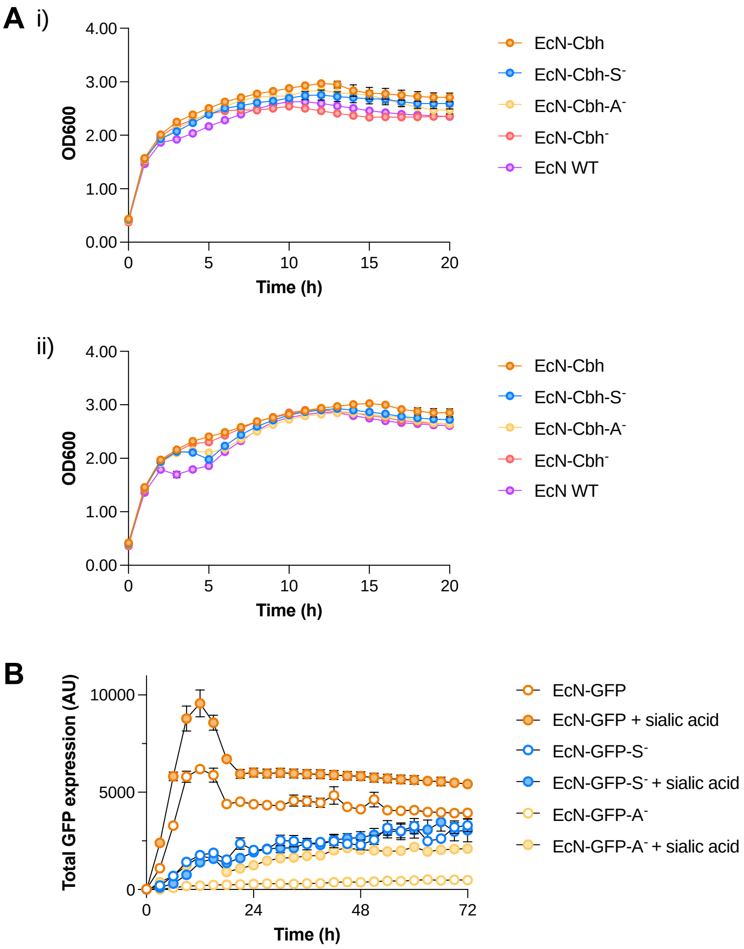

Supplementary Figure 5: Characterisation of the probiotic control groups. A) Growth profiles of probiotics i) without sialic acid and ii) with sialic acid induction. The error bars represent the SEMs of triplicates. The details of the probiotics are outlined in Figure 5B. B) Total GFP expression of the EcN-expressing full construct (J23113-*nanR*-pNanA-*cadC*-pCadBA-*gfp*; *EcN-GFP*), no-sensor construct (J23100-gfp; *EcN-GFP S*$^-$), and no-amplifier construct (J23113-*nanR*-pNanA-*gfp*; *EcN-GFP A*$^-$) with or without sialic acid induction over 72 hours. The error bars represent the SEMs of triplicates. The no-actuator construct used the same circuit design as the full construct and is not characterised here. GFP replaced Cbh as an expression protein for reporter purposes.

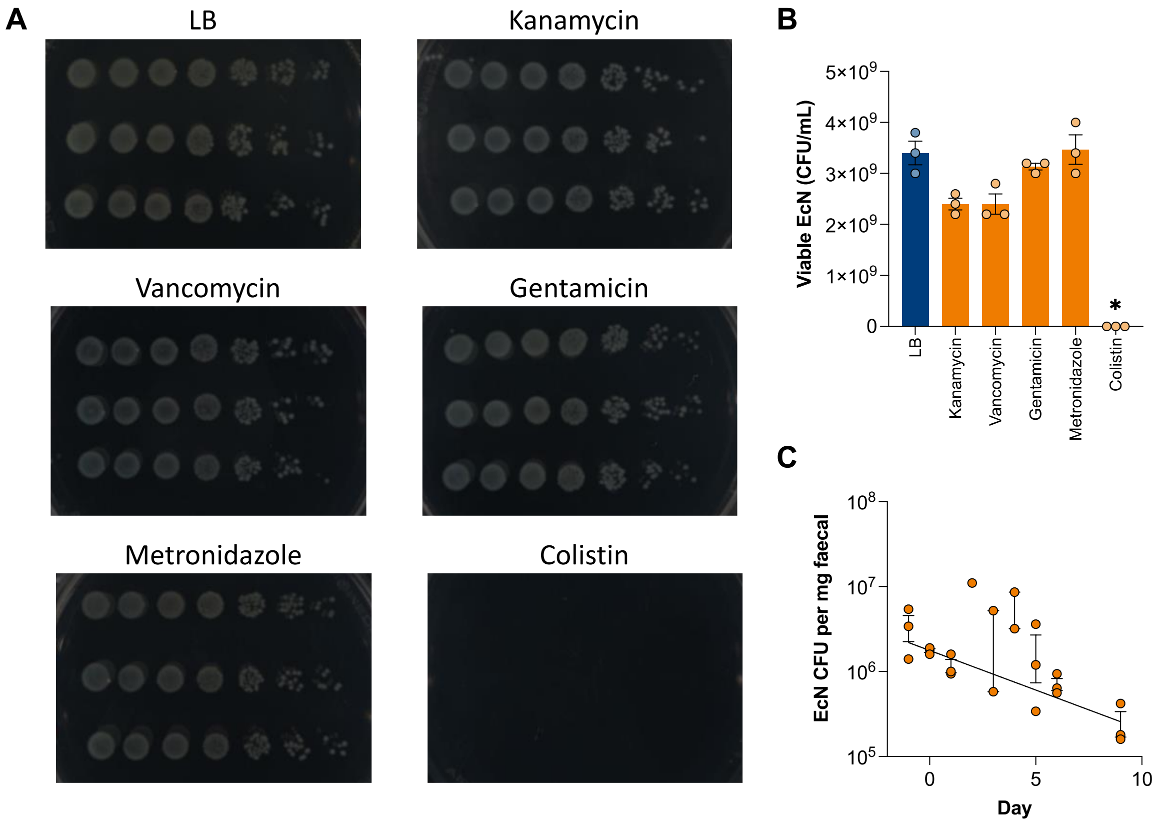

Supplementary Figure 6. In vitro and in vivo antimicrobial sensitivity of engineered probiotics. A) Image and B) enumeration of viable engineered probiotics, EcN-Cbh, after exposure to antibiotics at concentrations found in the cocktail (0.4 mg/mL kanamycin, 0.035 mg/mL gentamicin, 850 U/mL colistin, 0.215 mg/mL metronidazole, and 0.045 mg/mL vancomycin.) * No viable cells observed. C) Enumeration of engineered probiotics, EcN-Cbh, in faecal samples collected from mice given probiotics, after the antibiotic cocktail treatment. Data are presented as mean values with error bars representing SEMs of triplicates.

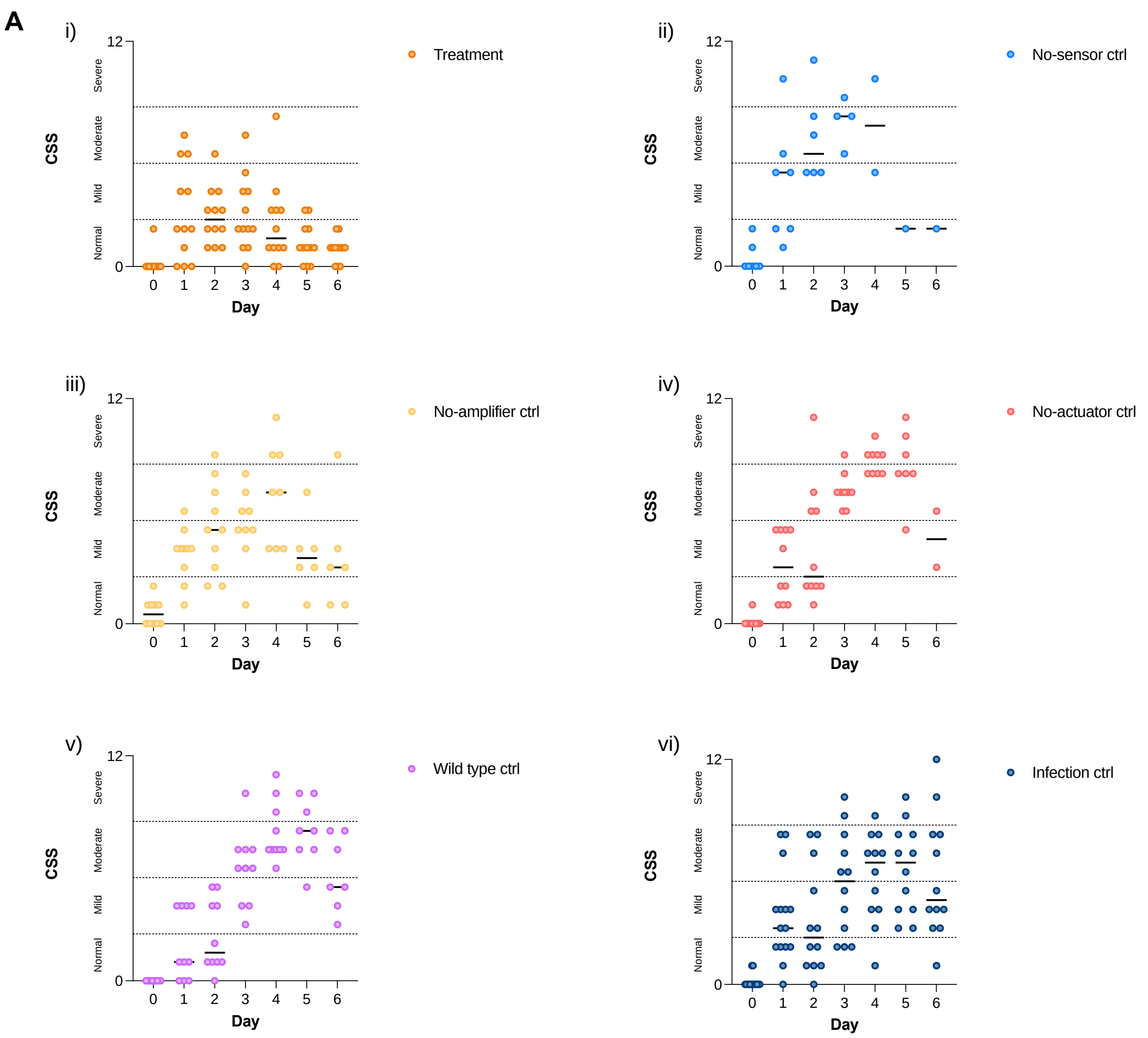

Supplementary Figure 7. Clinical Sickness Scores (CSS) for all treatment group over time. The CSS of each animal was recorded daily for 6 days post infection. The infection severity based on the score is indicated by dotted lines and corresponds to the following scale: normal, 0 to 2; mild, 3 to 5; moderate, 6 to 8; and severe, 9 to 12.

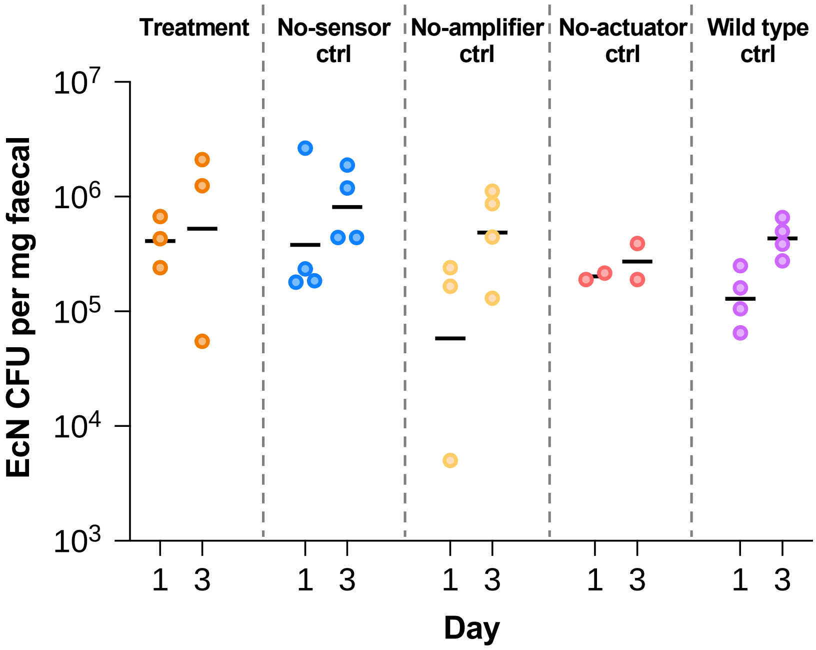

Supplementary Figure 8. Probiotics in mice with *C. difficile* infection. Enumeration of viable engineered or wild-type probiotics in faecal samples collected from mice on day 1 and day 3 post *C. difficile* infection.

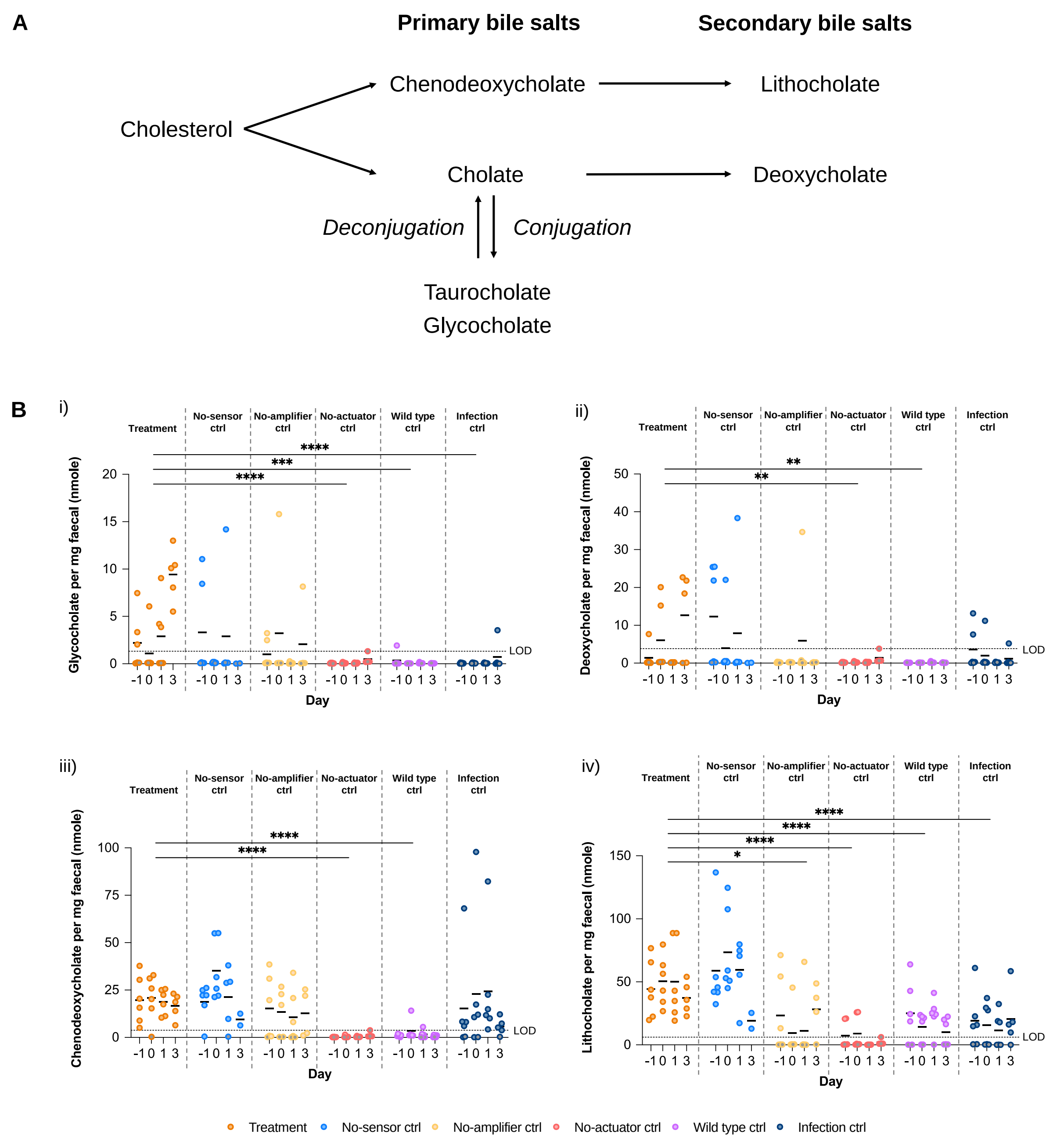

Supplementary Figure 9. Characterization of other bile salts in faecal samples. A) Overview of primary and secondary bile salts. B) Quantification of i) glycocholate, ii) deoxycholate, iii) chenodeoxycholate, and iv) lithocholate in faecal samples collected from day -1 to day 3 from each group. Mixed-model ANOVA was performed to compare groups. (p-value for column factor for glycocholate for no-sensor control group = 0.1447; no-amplifier control group = 0.0814; no-actuator control group < 0.0001; wild-type control group = 0.0001; infection control group < 0.0001; for deoxycholate for no-sensor control group = 0.7728; no-amplifier control group = 0.1378; no-actuator control group = 0.0059; wild-type control group = 0.0018; infection control group = 0.0574; for chenodeoxycholate for no-sensor control group = 0.5791; no-amplifier control group = 0.2814; no-actuator control group < 0.0001; wild-type control group < 0.0001; infection control group = 0.7819; for lithocholate for no-sensor control group = 0.4368; no-amplifier control group = 0.0138; no-actuator control group < 0.0001; wild-type control group < 0.0001; infection control group < 0.0001). * P < 0.05; ** P < 0.01; *** P < 0.001; **** P < 0.0001. The black bars indicate the means of the groups and days. LOD: limit of detection.
