# Supplementary material for: Engineering probiotics to inhibit Clostridioides difficile infection by dynamic regulation of intestinal metabolism
Source: Nat Commun. 2022 Jul 4;13:3834. doi: 10.1038/s41467-022-31334-z (PMC9253155; doi:10.1038/s41467-022-31334-z)
Supplement: Supplementary file 2 — Reporting Summary [file 41467_2022_31334_MOESM2_ESM.pdf]

nature portfolio

Corresponding author(s): Chang, Matthew Wook

Last updated by author(s): May 31, 2022

Reporting Summary

Nature Portfolio wishes to improve the reproducibility of the work that we publish. This form provides structure for consistency and transparency in reporting. For further information on Nature Portfolio policies, see our Editorial Policies and the Editorial Policy Checklist.

Statistics

For all statistical analyses, confirm that the following items are present in the figure legend, table legend, main text, or Methods section.

n/a	Confirmed	
☐	☒	The exact sample size (*n*) for each experimental group/condition, given as a discrete number and unit of measurement
☐	☒	A statement on whether measurements were taken from distinct samples or whether the same sample was measured repeatedly
☐	☒	The statistical test(s) used AND whether they are one- or two-sided<br>*Only common tests should be described solely by name; describe more complex techniques in the Methods section.*
☒	☐	A description of all covariates tested
☐	☒	A description of any assumptions or corrections, such as tests of normality and adjustment for multiple comparisons
☐	☒	A full description of the statistical parameters including central tendency (e.g. means) or other basic estimates (e.g. regression coefficient) AND variation (e.g. standard deviation) or associated estimates of uncertainty (e.g. confidence intervals)
☐	☒	For null hypothesis testing, the test statistic (e.g. *F*, *t*, *r*) with confidence intervals, effect sizes, degrees of freedom and *P* value noted<br>*Give P values as exact values whenever suitable.*
☒	☐	For Bayesian analysis, information on the choice of priors and Markov chain Monte Carlo settings
☒	☐	For hierarchical and complex designs, identification of the appropriate level for tests and full reporting of outcomes
☒	☐	Estimates of effect sizes (e.g. Cohen's *d*, Pearson's *r*), indicating how they were calculated

Our web collection on statistics for biologists contains articles on many of the points above.

Software and code

Policy information about availability of computer code

Data collection	Biotek Gen5, Agilent Chemstation, Amersham Imager 600 control software, Agilent MassHunter, Leica Las X, BD Accuri C6 Software
Data analysis	Microsoft Excel (version 16.60), Prism GraphPad, Qiime2, FlowJo

For manuscripts utilizing custom algorithms or software that are central to the research but not yet described in published literature, software must be made available to editors and reviewers. We strongly encourage code deposition in a community repository (e.g. GitHub). See the Nature Portfolio guidelines for submitting code & software for further information.

Data

Policy information about availability of data

All manuscripts must include a data availability statement. This statement should provide the following information, where applicable:
- Accession codes, unique identifiers, or web links for publicly available datasets
- A description of any restrictions on data availability
- For clinical datasets or third party data, please ensure that the statement adheres to our policy

Microbiome 16s rRNA sequencing data that support the findings of this study have been deposited in the Sequence Read Archive (SRA) (accession no: PRJNA844050). All other data supporting the findings of this study are available within the article and its supplementary information files.

Field-specific reporting

Please select the one below that is the best fit for your research. If you are not sure, read the appropriate sections before making your selection.

[x] Life sciences [ ] Behavioural & social sciences [ ] Ecological, evolutionary & environmental sciences

For a reference copy of the document with all sections, see nature.com/documents/nr-reporting-summary-flat.pdf

Life sciences study design

All studies must disclose on these points even when the disclosure is negative.

Sample size	For animal model, we used the formula n=1+2C(s/d)^2, where n is group sample size, s is standard deviation, d is the difference between mean of groups. For initial calculation, we assume s = 5 and d = 10 (based on Sun et al, 2011), to an estimated minimum sample size of 7 per group
Data exclusions	Animals that failed to exhibit sufficient infection symptoms were excluded to ensure that the efficacy of treatment is evaluated in mice groups with assured infection.
Replication	In vitro and in vivo experiments were repeated multiple times, at least twice independently as stated in figure legends. All animal experiments were repeated in 3-5 mice per group. All attempts at replication were successful.
Randomization	Samples and mice were randomly allocated into treatment groups.
Blinding	Analysis of histopathological injuries were conducted blind by trained pathologist. Sickness scoring was not performed blind as scoring was performed as ongoing experiment.

Reporting for specific materials, systems and methods

We require information from authors about some types of materials, experimental systems and methods used in many studies. Here, indicate whether each material, system or method listed is relevant to your study. If you are not sure if a list item applies to your research, read the appropriate section before selecting a response.

Materials & experimental systems

n/a	Involved in the study	
[ ]	[x]	Antibodies
[ ]	[x]	Eukaryotic cell lines
[x]	[ ]	Palaeontology and archaeology
[ ]	[x]	Animals and other organisms
[x]	[ ]	Human research participants
[x]	[ ]	Clinical data
[x]	[ ]	Dual use research of concern

Methods

n/a	Involved in the study	
[x]	[ ]	ChIP-seq
[x]	[ ]	Flow cytometry
[x]	[ ]	MRI-based neuroimaging

Antibodies

Antibodies used	Mouse monoclonal to Clostridium difficile toxin A (PCG4, ab19953, Abcam) used at final concentration of 500µg/L.<br>Mouse monoclonal anti-His tag antibody conjugated with HRP at 1:1000 dilution (#9991S, Cell Signaling Technology).<br>Anti-mouse IgG conjugated to HRP at 1:2000 dilution (#7076, Cell Signaling Technology).
Validation	Manufacturer validation data for western blotting in Pubmed: 24244530

Eukaryotic cell lines

Policy information about cell lines

Cell line source(s)	Caco-2 (ATCC)
Authentication	Cell line authenticated by ATCC
Mycoplasma contamination	All cell line tested negative for mycoplasma
Commonly misidentified lines (See ICLAC register)	No commonly misidentified lines were used.

Animals and other organisms

Policy information about studies involving animals; ARRIVE guidelines recommended for reporting animal research

Laboratory animals	C57BL/6, 5-6 week-old, male
Wild animals	Not involved
Field-collected samples	Not involved
Ethics oversight	National University of Singapore Institutional Animal Care and Use Committee (Protocol R18-0329)

Note that full information on the approval of the study protocol must also be provided in the manuscript.
